# Induction Chemoimmunotherapy Followed by Consolidative Hypofractionated Radiotherapy for Unresectable Locally Advanced NSCLC: A Real-World Outcomes Analysis

**DOI:** 10.3390/cancers18132036

**Published:** 2026-06-23

**Authors:** Caglayan Selenge Beduk Esen, Sukran Celikarslan, Duygu Sezen, Fatih Selcukbiricik, Kerim Kaban, Metin Kanitez, Perran Fulden Yumuk, Nil Molinas Mandel, Levent Tabak, Ezgi Cesur, Suat Erus, Serhan Tanju, Sukru Dilege, Terman Gumus, Cetin Atasoy, Cengiz Demirkurek, Okan Falay, Mehmet Onur Demirkol, Pinar Bulutay, Pinar Firat, Melis Selek, Merve Duman, Sepideh Mohammadipour, Saliha Ezgi Oymak, Nulifer Kilic Durankus, Yasemin Atagun, Ugur Selek

**Affiliations:** 1 Department of Radiation Oncology, American Hospital, Istanbul 34365, Türkiye; caglayane@amerikanhastanesi.org (C.S.B.E.); smohammadipour@amerikanhastanesi.org (S.M.); ezgioy@amerikanhastanesi.org (S.E.O.); ybolukbasi@kuh.ku.edu.tr (Y.A.); 2 Department of Radiation Oncology, Koç University School of Medicine, Istanbul 34450, Türkiye; scelikarslan@kuh.ku.edu.tr (S.C.); dsezen@ku.edu.tr (D.S.); caglayanmerve.itf@gmail.com (M.D.); ndurankus@kuh.ku.edu.tr (N.K.D.); 3 Department of Medical Oncology, Koç University School of Medicine, Istanbul 34450, Türkiye; fatihse@amerikanhastanesi.org; 4 Department of Medical Oncology, American Hospital, Istanbul 34365, Türkiye; kerimk@amerikanhastanesi.org (K.K.); metinka@amerikanhastanesi.org (M.K.); fyumuk@kuh.ku.edu.tr (P.F.Y.); nilm@amerikanhastanesi.org (N.M.M.); 5 Department of Pulmonary Medicine, American Hospital, Istanbul 34365, Türkiye; leventt@amerikanhastanesi.org; 6 Department of Thoracic Surgery, American Hospital, Istanbul 34365, Türkiye; ezgic@amerikanhastanesi.org (E.C.); sukrud@amerikanhastanesi.org (S.D.); 7 Department of Thoracic Surgery, Koç University School of Medicine, Istanbul 34450, Türkiye; suate@amerikanhastanesi.org (S.E.); serhant@amerikanhastanesi.org (S.T.); 8 Department of Radiology, American Hospital, Istanbul 34365, Türkiye; termangu@amerikanhastanesi.org; 9 Department of Radiology, Koç University School of Medicine, Istanbul 34450, Türkiye; catasoy@kuh.ku.edu.tr; 10 Department of Nuclear Medicine, American Hospital, Istanbul 34365, Türkiye; cengizd@amerikanhastanesi.org (C.D.); onurd@amerikanhastanesi.org (M.O.D.); 11 Department of Nuclear Medicine, Koç University School of Medicine, Istanbul 34450, Türkiye; okanf@amerikanhastanesi.org; 12 Department of Pathology, Koç University School of Medicine, Istanbul 34450, Türkiye; pinarbul@amerikanhastanesi.org (P.B.); pfirat@kuh.ku.edu.tr (P.F.); 13 Koç University School of Medicine, Istanbul 34450, Türkiye; mselek22@ku.edu.tr

**Keywords:** non-small cell lung cancer, immunotherapy, hypofractionated radiotherapy, survival, toxicity

## Abstract

**Purpose/Objective:** To evaluate the safety and failure patterns of consolidative hypofractionated thoracic radiotherapy (RT) following induction chemoimmunotherapy in patients with unresectable locally advanced non-small cell lung cancer (LA-NSCLC). **Materials/Methods:** This retrospective study included 34 patients treated between 2019 and 2025. All patients received induction chemoimmunotherapy followed by consolidative hypofractionated RT based on multidisciplinary tumor board recommendations. The primary endpoint was local recurrence (LR); secondary endpoints were regional recurrence (RR), distant metastasis (DM), overall survival (OS), progression-free survival (PFS), and treatment-related toxicity. **Results:** Median age was 64 years, and median PD-L1 expression was 20%. Most patients had stage III disease; squamous cell carcinoma (56%) and adenocarcinoma (38%) were the predominant histologies. The most common systemic regimen was carboplatin/paclitaxel plus nivolumab, with a median of four induction cycles. Post-induction response was complete in 21%, partial in 62%, stable in 12%, and progressive in 6%. Median RT dose was 52.5 Gy in 15 fractions, and maintenance immunotherapy was administered in 79%. At a median follow-up of 16.7 months, using cumulative incidence functions with death treated as a competing event, the 1- and 2-year cumulative incidences of local failure were 6.9% and 14.7%, respectively. The corresponding cumulative incidences of regional failure were 10.2% and 18.8%, while distant metastasis incidences were 15.9% and 39.2%. No isolated local or regional recurrences occurred. One- and two-year OS rates were 86% and 81%, and corresponding PFS rates were 76% and 54%. No grade 4–5 RT-related toxicity occurred; one grade 5 immune-related pneumonitis was observed. **Conclusions:** Consolidative hypofractionated RT following chemoimmunotherapy appears feasible and associated with favorable outcomes, supporting further prospective investigation.

## Simple Summary

This real-world study evaluated hypofractionated thoracic radiotherapy after induction chemoimmunotherapy in patients with unresectable locally advanced non-small cell lung cancer. The treatment was associated with favorable locoregional control, encouraging survival outcomes, and acceptable toxicity. These findings support further prospective investigation of this sequential treatment strategy.

## 1. Introduction

Lung cancer remains the leading cause of cancer-related mortality worldwide, and approximately one-third of patients present with locally advanced non-small cell lung cancer (LA-NSCLC) [1,2]. Despite major therapeutic advances, prognosis for unresectable locally advanced disease remains suboptimal [3]. Concurrent chemoradiotherapy (CRT) followed by consolidation durvalumab, established by the PACIFIC trial, has become the standard of care and significantly improved survival outcomes compared with historical CRT alone [4,5]. However, distant metastasis remains a predominant mode of treatment failure, highlighting the need for strategies that improve systemic disease control while maintaining durable locoregional control.

Recent advances in neoadjuvant therapy have raised interest in moving immunotherapy earlier in the treatment sequence. In resectable NSCLC, phase III studies including CheckMate 816, KEYNOTE-671, and AEGEAN have demonstrated that induction chemoimmunotherapy can enhance tumor response and improve long-term outcomes [6,7,8,9,10]. Extending this paradigm to unresectable LA-NSCLC may offer similar advantages, including earlier eradication of micrometastatic disease and immune priming before definitive local therapy. Emerging prospective studies have explored induction chemoimmunotherapy followed by thoracic radiotherapy, with encouraging efficacy signals, although substantial toxicity has been observed in regimens incorporating concurrent chemoradiotherapy [11,12,13]. Thus, the optimal integration of induction systemic therapy and thoracic radiation remains undefined.

Hypofractionated radiotherapy (HFRT) may offer a particularly attractive consolidative platform in this setting [14,15,16]. Beyond shortening treatment duration, HFRT has demonstrated favorable efficacy and tolerability in LA-NSCLC and may possess unique immunomodulatory properties [17]. Preclinical data suggest moderate hypofractionation may enhance immunogenic cell death, tumor antigen release, and T-cell priming, potentially augmenting synergy with immune checkpoint blockade [17,18,19]. This concept aligns with the emerging paradigm of immune priming before definitive local therapy, whereby induction chemoimmunotherapy may expand tumor-specific T-cell populations and improve systemic antitumor immunity prior to radiotherapy. In this context, sequential HFRT following induction chemoimmunotherapy may represent a biologically favorable strategy to further amplify antitumor immune responses while avoiding the toxicity associated with concurrent chemoradiotherapy.

Real-world clinical data evaluating this sequential approach remain limited, particularly using consolidative thoracic HFRT delivered with modern image-guided techniques. In addition, patterns of failure and treatment-related toxicity associated with this strategy have not been well characterized. We hypothesized that this sequential strategy could improve distant disease control while preserving excellent locoregional control with acceptable toxicity. To test this hypothesis, we conducted a retrospective analysis evaluating oncologic outcomes, failure patterns, and treatment-related adverse events in patients treated with this approach.

## 2. Materials and Methods

### 2.1. Patient Selection

**Figure 1:**
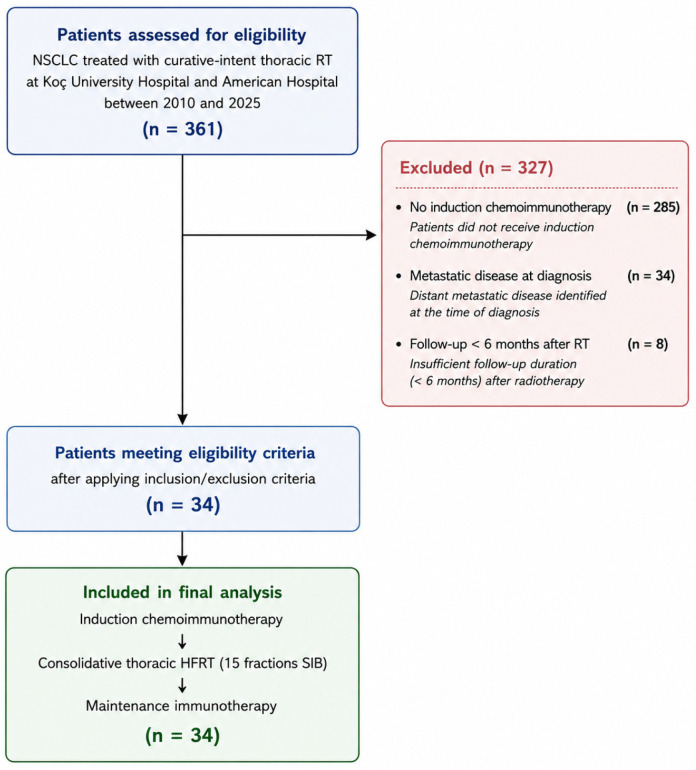
STROBE Flow Diagram of Patient Selection. NSCLC, non-small cell lung cancer; RT, radiotherapy; HFRT, hypofractionated radiotherapy; SIB, simultaneous integrated boost.

The medical records of 361 patients diagnosed with NSCLC and treated with curative intent RT between 2010 and 2025 at Koç University Hospital and American Hospital were retrospectively reviewed. The inclusion criteria were: (1) age ≥ 18 years; (2) pathologically confirmed NSCLC; (3) no evidence of distant metastasis (DM) at the time of diagnosis; (4) receipt of consolidative thoracic HFRT; and (5) a minimum follow-up of 6 months after RT (Figure 1). Patients were initially treated with induction chemoimmunotherapy with the intent of reassessing surgical eligibility after treatment response. Following multidisciplinary tumor board (MTB) reevaluation, patients who remained unsuitable for resection due to persistent nodal disease, inadequate response, inadequate pulmonary reserve, technical unresectability, surgeon assessment, and/or medical inoperability proceeded to consolidative thoracic HFRT. Patients were excluded if they had not received induction chemoimmunotherapy before consolidative thoracic RT, or if they received thoracic stereotactic body RT (SBRT) or CFRT instead of thoracic HFRT. No patients with known actionable oncogenic driver alterations were identified in the final study cohort. After applying these criteria, 34 patients with LA-NSCLC treated with induction chemoimmunotherapy followed by consolidative thoracic HFRT and maintenance immunotherapy were included in the final analysis. Immunotherapy was integrated into treatment protocols at our institutions in 2019. The mixed prescription nature of immunotherapy agents in this cohort was directly related with accessibility, and/or insurance approval policy at the time of the discretion. Therefore, this study focused on patients treated between 2019 and 2025, reflecting current real-world practice. This retrospective study was administered in accordance with the principles of the Helsinki Declaration. This study was approved by the Institutional Review Board of Koç University (IRB number: 2025.490.IRB2.223). Written informed consent was obtained from all patients.

### 2.2. Treatment

All patients received consolidative thoracic HFRT after induction chemoimmunotherapy and MTB review determined the disease to be unresectable. The number of chemoimmunotherapy cycles was determined at the discretion of the treating physician based on individual treatment response and tolerability. The maintenance immunotherapy was administered in patients without severe treatment-related toxicity until disease progression or any unacceptable toxicity.

All patients underwent ^18^F-fluorodeoxyglucose (FDG) positron emission computed tomography (PET-CT) and thorax CT imaging at diagnosis and following completion of induction chemoimmunotherapy to assess locoregional tumor extent, rule out distant metastases (DM), and evaluate treatment response. All FDG PET-CT images were carefully evaluated by a nuclear medicine specialist according to iPERCIST criteria [20].

Four-dimensional CT (4DCT) was utilized for the RT planning, with target volumes contoured on the average respiratory phase to include tumor motion across all respiratory phases. RT planning was performed using a simultaneous integrated boost (SIB) approach with intensity-modulated radiotherapy (IMRT) techniques [21]. Lung dose was evaluated on the expiratory phase (V50), while esophageal dose constraints were based on a composite volume including full motion. Daily cone-beam CT was used to minimize setup errors.

The gross tumor volume (GTV) was defined as the primary tumor and involved lymph nodes (LNs) following induction chemoimmunotherapy. The high-risk clinical target volume (CTV-HR) was generated by adding a 3 mm margin to the GTV, while the low-risk clinical target volume (CTV-LR) encompassed the initially involved nodal regions. PTV was generated by adding a 3 mm margin to CTVs.

Concurrent chemotherapy was intentionally omitted during RT to minimize treatment-related toxicity. Immunotherapy, however, was permitted either during or after RT according to physician discretion. After completion of induction chemoimmunotherapy, further cytotoxic therapy during RT was avoided. In this context, consolidative HFRT delivered in 15 fractions [16,22] was selected to provide effective locoregional treatment while potentially enhancing immune activation without the additional burden of concurrent chemotherapy. Accordingly, the prescribed total doses were 60 Gy to the GTV, 52.5 Gy to the CTV-HR, and 45 Gy to the CTV-LR, all administered over 15 fractions using a SIB approach.

### 2.3. Follow-Up

Treatment response was assessed six weeks after the completion of RT using a thoracic CT scan. Patients were subsequently followed up every three months during the first two years, every six months from the third to the fifth year, and annually thereafter. Local recurrence (LR) was defined as progression of the primary tumor; regional recurrence (RR) as progression in mediastinal LNs; and DM as metastatic spread to distant organs. Patients who developed oligoprogressive disease during follow-up were considered for local ablative treatment, including surgery or SBRT depending on resectability and patient suitability. Patients with multiple metastatic sites or disseminated progression were treated with systemic therapy according to multidisciplinary evaluation. In cases where recurrence was clinically suspected, FDG PET-CT was performed for further evaluation.

All treatment-related toxicities were graded according to the Common Terminology Criteria for Adverse Events (CTCAE) version 5.0. Adverse events occurring within 90 days of treatment were considered acute, whereas those occurring after 90 days were classified as late toxicities. Pneumonitis events were retrospectively reviewed according to imaging findings and their relationship to the irradiated lung volumes. Events occurring predominantly within the RT treatment fields were classified as radiation pneumonitis, whereas events occurring outside the treatment fields were classified as immunotherapy-related pneumonitis.

### 2.4. Statistical Analyses

Statistical analyses were performed using the Statistical Package for Social Sciences (SPSS) version 24.0 (IBM Corp., Armonk, NY, USA). The primary endpoint was local control (LC), while the secondary endpoints included RR, DM, OS, PFS, and treatment-related adverse events (TRAEs). Age, gender, smoking status, histopathology, PD-L1 score (TPS), pan-immune-inflammation-value (PIV), TNM stage (9th edition), the number of total immunotherapy cycles, the time interval between the diagnosis and RT initiation, and the intent to RT were determined as covariates. PIV was calculated using peripheral blood neutrophil (N), platelet (P), lymphocyte, and monocyte (M) counts obtained at the diagnosis with the formula of PIV = P × M × N ÷ L.

All time-to-event outcomes were measured from the diagnosis until the date of last follow-up, death, or recurrence, whichever occurred first. Synchronous failures were defined as locoregional and distant failures detected at the same radiologic assessment or within 30 days of each other. Cumulative incidence functions were used to estimate local, regional, and distant failure, with death without prior recurrence treated as a competing event. Kaplan–Meier analysis was used to estimate OS and PFS rates. The prognostic factors for OS and PFS were analyzed using the log-rank test. Exploratory univariate analyses were performed. A *p*-value < 0.05 was considered statistically significant.

## 3. Results

### 3.1. Patient Characteristics

Patient and tumor characteristics are detailed in Table 1. The most common chemotherapy regimen was carboplatin plus paclitaxel (74%) followed by carboplatin plus pemetrexed (8%), cisplatin plus pemetrexed (6%), gemcitabine plus cisplatin (6%), carboplatin plus pemetrexed with bevacizumab (3%), and carboplatin only (3%). The frequently used immunotherapy regimen was nivolumab (59%) followed by pembrolizumab (25%), atezolizumab (8%), and durvalumab (8%). The median number of chemotherapy and immunotherapy induction cycles was 4 (range: 2–6) and 4 (range: 2–9), respectively. All patients received at least three cycles of chemoimmunotherapy, except for one patient who received two cycles due to immunotherapy-related pneumonia. Following induction therapy, 7 (21%) and 21 (62%) patients achieved a complete and partial response, respectively, and 4 (12%) and 2 (6%) had stable disease and disease progression, respectively. The intent to RT was consolidation in 29 (85%) patients and progression in 5 (15%). “Progression intent RT” was defined as thoracic HFRT delivered to patients who experienced disease progression during induction chemoimmunotherapy but remained free of DM and unsuitable for surgical resection. The median duration from the diagnosis to initiation of RT was 4.46 months (range: 2.66–86.57 months). The median prescribed RT dose was 52.5 Gy (range: 45–60 Gy) in 15 fractions. Immunotherapy was delivered concomitantly with RT in 14 (41%) patients; however, no patient received concurrent chemotherapy during RT, as specified by the study protocol. Twenty-seven (79%) patients received maintenance immunotherapy after RT, with a median of 9 cycles (range: 1–63). The median total number of immunotherapy cycles was 11 (range: 2–69).

### 3.2. Oncologic Outcomes

The median follow-up time for the entire cohort was 16.7 months (IQR: 10.5–31.0 months). At the time of the last follow-up, 23 (68%) patients were alive without evidence of disease, 3 (9%) patients were alive with disease, 6 (17%) died due to progressive disease, and 2 (6%) died due to causes other than lung cancer. Using cumulative incidence functions with death treated as a competing event, the 1- and 2-year cumulative incidences of local failure were 6.9% and 14.7%, respectively. The corresponding cumulative incidences of regional failure were 10.2% and 18.8%, while distant metastasis incidences were 15.9% and 39.2%. Overall, 4 patients experienced local failure, 4 experienced regional failure, and 10 developed distant metastatic recurrence. No isolated local or regional recurrences occurred. The most common sites of distant metastatic recurrence were lung parenchyma (55%) followed by brain (27%), bone (18%), and adrenal gland (18%).

**Figure 2:**
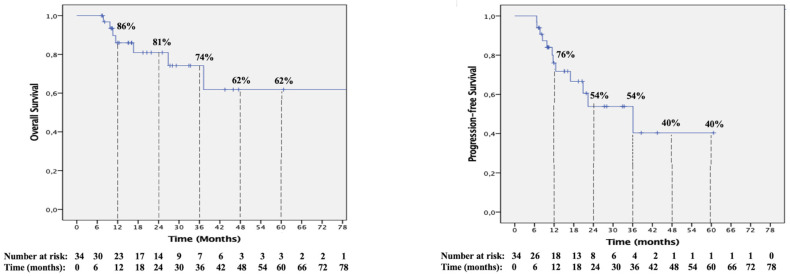
Kaplan–Meier survival estimates of overall survival and progression-free survival.

The median OS was not reached, while the median PFS was 36.1 months (95% CI, 14.8–57.3). The OS rates at 1 and 2 years were 86% and 81%, respectively, while the corresponding PFS rates were 76% and 54%. These outcomes appear encouraging, within the context of this selected retrospective cohort, as illustrated in Figure 2.

No covariates were significantly associated with OS or PFS (Table 2). Although no statistically significant differences were observed, a consistent numerical trend toward poorer OS and PFS was noted in patients who received RT with the intent of disease progression compared with those with the intent of consolidation (82% vs. 75%, *p* = 0.881 for 2-y OS and 63% vs. 25%, *p* = 0.320 for 2-y PFS). ROC analysis identified a PIV threshold of 1381.5, yielding a sensitivity of 46% and a specificity of 91% (AUC = 0.709, *p* = 0.043). Similarly, when patients were stratified according to PIV (<1381.5 vs. ≥1381.5), those with higher PIVs demonstrated numerically worse OS and PFS, although this difference did not reach statistical significance (*p* = 0.121).

### 3.3. Toxicity

The treatment was well tolerated, and no radiation-related deaths were reported. Radiation-induced grade 1–2 and grade 3 acute esophagitis occurred in 24 (71%) and 3 (9%) patients, respectively, with no grade 4 or 5 events. Grade 2 and grade 3 radiation-induced pneumonitis occurred in 2 (6%) and 1 (3%) patients, respectively. One patient developed both grade 3 thyroiditis and pancreatitis, which were attributed to immunotherapy. Immunotherapy-related pneumonitis of grade 2, grade 3, and grade 5 was observed in 1 (3%), 3 (9%), and 1 (3%) patients, respectively. The grade 5 pneumonitis event occurred in a patient who received immunotherapy concomitantly with RT. Two (6%) patients discontinued treatment due to immunotherapy-related pneumonitis.

## 4. Discussion

In this retrospective analysis, induction chemoimmunotherapy followed by consolidative thoracic HFRT and maintenance immunotherapy was associated with favorable locoregional control, encouraging survival outcomes, and an acceptable toxicity profile in selected patients with unresectable LA-NSCLC. With a median OS not reached and 2-year OS and PFS rates of 81% and 54%, respectively, our results appear encouraging in the context of historical benchmarks reported for standard CRT-based approaches. However, these findings should be interpreted with caution, as concurrent chemoradiotherapy followed by consolidation durvalumab remains the established standard of care for eligible patients with unresectable stage III NSCLC. Given the retrospective nature of this study and the substantial differences in patient selection, treatment sequencing, and study design, no direct comparison with contemporary standard-of-care regimens can be made. Notably, isolated locoregional failures were not observed, and distant metastasis represented the predominant mode of progression, consistent with evolving patterns of failure in the immunotherapy era. Collectively, these findings suggest that this sequential strategy may represent a feasible consolidation paradigm deserving prospective evaluation rather than an alternative to established standard-of-care approaches at present.

Interest in induction chemoimmunotherapy for unresectable locally advanced NSCLC has grown as a strategy intended to improve systemic control while potentially enhancing response to subsequent local therapy. Prospective studies such as KEYNOTE-799, AFT-16, and APOLO have demonstrated encouraging efficacy signals, albeit with substantial toxicity in intensified concurrent approaches (Table 3) [11,12,13]. Retrospective data also support further exploration of this sequential paradigm. Kleber et al. reported variable outcomes across CFRT, HFRT, and SBRT following induction chemoimmunotherapy, although interpretation was limited by incomplete reporting of dose-fractionation and stage distribution [22]. Notably, symptomatic pneumonitis rates in that series were higher than those observed in our cohort. Similarly, Mo et al. reported comparable efficacy with improved safety for induction chemoimmunotherapy followed by sequential CFRT versus conventional concurrent CRT plus durvalumab [23]. Tang et al. likewise demonstrated encouraging tolerability with induction chemoimmunotherapy followed by thoracic RT and maintenance immunotherapy, with low rates of severe toxicity [24]. In this context, our findings and emerging retrospective data suggest sequential chemoimmunotherapy followed by consolidative radiation may represent a potentially more tolerable intensification strategy deserving prospective evaluation. It should be noted that induction chemoimmunotherapy may facilitate conversion to resectability in a subset of patients with initially unresectable LA-NSCLC, thereby potentially altering the subsequent treatment paradigm. However, all patients included in the present cohort remained unsuitable for surgical resection following multidisciplinary evaluation and were therefore treated with definitive radiotherapy-based therapy.

A potentially distinguishing feature of this strategy is the use of consolidative HFRT [25]. Beyond shortening treatment duration, moderate hypofractionation may offer biologic synergy with immunotherapy through enhanced immunogenic cell death and T-cell priming [26,27]. It is conceivable that induction chemoimmunotherapy may establish a primed antitumor immune milieu that can subsequently be amplified by hypofractionated radiotherapy. In this framework, sequential HFRT may serve not only as a local consolidative treatment but also as a potential immunomodulatory stimulus capable of reinforcing systemic antitumor immunity. Consistent with this rationale, Zhu et al. reported encouraging survival and manageable toxicity with induction chemoimmunotherapy followed by HFRT in unresectable stage III disease [28]. Together with the favorable locoregional control observed here, these findings support prospective evaluation of HFRT as a consolidation platform in radioimmunotherapy strategies.

**Table 3:** Studies evaluating induction chemoimmunotherapy followed by radiotherapy in patients with locally advanced non-small cell lung cancer.

Prospective Studies	N	Patient Selection	RT Dose, Fractionation	Median Follow-Up (Months)	Outcomes	Toxicity
KEYNOTE-799 [11]	214	Unresectable stage III NSCLC Cohort A and B (SCC/non-SCC and non-SCC)	CFRT with concurrent chemotherapy (60 Gy/30 fr)	59.2 and 54.4 (cohort A and B)	Treatment discontinuation: 38 (33.9%) and 21 (20.6%) pts (cohort A and B) ORR: 71.4% and 75.5% (cohort A and B) 4-y OS and PFS: 40.2% and 38.9% (cohort A) 4-y OS and PFS: 54.7% and 46.3% (cohort B)	Grade ≥ 3 TRAE 65.2% and 51.0% (cohort A and B)
AFT-16 [12]	62	Unresectable stage III NSCLC	CFRT with concurrent chemotherapy (60 Gy/30 fr)	31.2	Treatment discontinuation: 12 (19.4%) ORR: 66.2% 12- and 24-mo OS: 87.0% and 73.7% 12- and 24-mo PFS: 68.9% and 54.2%	Grade ≥ 3 TRAE ≥ 48.4% Grade 5 TRAE 3.2%
APOLO [13]	38	Unresectable stage III NSCLC	CFRT with concurrent chemotherapy (66 Gy/30 fr)	29.6	Treatment discontinuation: 5 (13.1%) 12- and 24 mo OS: 86.8% and 60.5% 12- and 24 mo PFS: 68.4% and 50.0%	Grade ≥ 3 TRAE ≥ 55.2% Grade 5 TRAE 2.6%
Retrospective studies						
Kleber et al. [22]	49	LA-NSCLC (Stage I–III)	CFRT vs. HFRT vs. SBRT (details NA) No concurrent chemotherapy	NA	Treatment discontinuation: NA Overall LC: 93%, 83%, and 100% 12-mo OS: 84%, 58%, 100% 12-mo PFS: 61%, 51%, 75%	Symptomatic pneumonitis: 33%, 21%, 20% (CFRT, HFRT, SBRT)
Mo et al. [23]	102	Unresectable stage III NSCLC	CFRT (60 Gy/30 fr) (sequential vs. concurrent chemotherapy)	27	Treatment discontinuation: NA (sequential vs. concurrent) ORR: 74.2% vs. 63.9% 1-, 3-, and 5-y OS: 98.5%, 61.4%, and 52.1% vs. 97.2%, 38.3%, and NA (*p* = 0.52) 1-, 3-, 5-y PFS: 81.4%, 47.1%, and 47.1% vs. 73%, 38.7%, and not reached. (*p* = 0.71)	(sequential vs. concurrent) Grade ≥ 3 TRAE ≥ 15.1% vs. 8.4% No grade 5 TRAE
Tang et al. [24]	30	Unresectable stage III NSCLC	CFRT (details NA)	16.3	Treatment discontinuation: NA ORR: 86.7% 12-mo PFS: 86.7%	Grade ≥ 3 TRAE 20% No grade 5 TRAE
Zhu et al. [28]	35	Unresectable stage III NSCLC	HFRT (44–68 Gy/15 fr)	31.5	Treatment discontinuation: NA 1-, 2-, and 3-y OS: 100%, 82.5%, and 77.3% 1-, 2-, and 3-y PFS: 74.3%, 55.7%, and 47.6%	Grade ≥ 3 TRAE 14.2% (1 grade 4 immune-pneumonitis; 1 grade 3 RT-pneumonitis)
Current study	34	Unresectable LA-NSCLC	HFRT (60/52.5/45 Gy/15 fr, SIB)	16.7	Treatment discontinuation: 6% 1- and 2-y LC: 94%, 88% 1-, 2-, and 5-y OS: 86%, 81%, and 62% 1-, 2-, and 5-y PFS: 76%, 54%, and 40%	No grade 4–5 RT toxicity; 1 grade 5 immune pneumonitis

**Abbreviations:** RT = Radiotherapy; NSCLC = Non-small cell lung cancer; LA-NSCLC = Locally advanced non-small cell lung cancer; SCC = Squamous cell carcinoma; CFRT = Conventional fractionated radiotherapy; HFRT = Hypofractionated radiotherapy; SBRT = Stereotactic body radiation therapy; ORR = Objective response rate; OS = Overall survival; PFS = Progression-free survival; LC = Local control; TRAE = Treatment-related adverse events; SIB = Simultaneous-integrated boost.

Importantly, treatment was associated with an acceptable safety profile, with low rates of severe radiation-related toxicity and limited treatment discontinuation. The sequential treatment design was intentionally selected to avoid the cumulative toxicity associated with concurrent chemoradiotherapy-based approaches. In addition, the optimal integration of chemotherapy, radiotherapy, and immune checkpoint blockade remains incompletely defined, and concerns have been raised that excessive treatment intensification may adversely affect treatment tolerability and potentially influence immune-mediated antitumor responses. This may reflect, in part, the sequential treatment design avoiding concurrent chemotherapy during radiation, as well as the use of modern image-guided planning, motion management, and a uniform hypofractionated SIB approach. Although one grade 5 immune-related pneumonitis event occurred in a patient who received immunotherapy concomitantly with RT, severe toxicity overall appeared manageable and compares favorably with reports from several intensified combined-modality immunotherapy studies [29]. These observations suggest that this strategy may offer a feasible balance between intensification and tolerability, although prospective validation is needed.

Although no statistically significant prognostic factors for OS or PFS were identified, patients treated with RT for disease progression and those with elevated baseline PIV showed numerically poorer outcomes. Prior studies have associated lower PIV with improved survival in immunotherapy-treated NSCLC, suggesting systemic inflammatory burden may influence response to radioimmunotherapy [30]. While these observations were hypothesis-generating and limited by sample size, they suggest that PIV may warrant further investigation as an exploratory biomarker in this setting. Other candidate biomarkers that may help guide treatment selection and predict outcomes in radioimmunotherapy strategies include PD-L1 expression, circulating tumor DNA (ctDNA), and tumor mutational burden (TMB). In particular, dynamic ctDNA assessment has emerged as a promising tool for monitoring treatment response and minimal residual disease. Prospective studies incorporating biomarker-driven approaches may help refine patient selection for induction chemoimmunotherapy and consolidative radiation strategies.

Several limitations warrant acknowledgment. The retrospective design introduces potential selection bias, particularly given multidisciplinary selection of patients considered appropriate for this approach. The relatively small sample size and limited event numbers constrain definitive conclusions regarding efficacy and prognostic factors, and the absence of a control cohort precludes direct comparison with standard CRT-based approaches. Due to the retrospective nature of the study, detailed data regarding steroid dosing, hospitalization, and imaging follow-up were not uniformly available for all patients. Therefore, these variables could not be systematically evaluated and should be considered when interpreting the results. In addition, heterogeneity in systemic therapy regimens and maintenance immunotherapy may have influenced outcomes. Accordingly, these findings should be viewed as hypothesis-generating. Nevertheless, the use of a consistent departmental HFRT platform and detailed characterization of outcomes and failure patterns provide clinically relevant real-world evidence. Taken together, our results support induction chemoimmunotherapy followed by consolidative HFRT as a promising investigational consolidation paradigm that warrants prospective testing to better define patient selection, sequencing, and the role of hypofractionation in locally advanced NSCLC.

## 5. Conclusions

In this hypothesis-generating real-world cohort, induction chemoimmunotherapy followed by consolidative hypofractionated thoracic radiotherapy was associated with encouraging locoregional control with acceptable toxicity in selected patients with unresectable locally advanced NSCLC. The absence of isolated locoregional failure observed in this cohort suggests that distant progression may remain the predominant pattern of treatment failure, highlighting the continued importance of systemic disease control. Prospective studies are warranted to define the role of this sequential strategy relative to standard chemoradiotherapy-based approaches.

## Figures and Tables

**Table 1:** Patient and tumor characteristics.

Characteristics	n	%
Age (years, median, range)	64 (33–78)	
PD-L1 expression (%, median, range)	20 (0–95)	
Pan-immune inflammation index value (PIV) (median, range)	685.5 (137–4126)	
Smoking exposure, pack-years, median (IQR)	30 (9.3–46.3)	
**Gender**		
Female	10	29
Male	24	71
**Tobacco Use**		
Current smoker	17	50
Former smoker	11	32
Never smoker	6	18
**Number of Comorbidities**		
0	10	29
1	14	41
2	5	15
≥3	5	15
**Histopathology**		
Squamous cell carcinoma	19	56
Adenocarcinoma	13	38
Other	2	6
**T Stage (8th Edition)**		
T1b	1	3
T1c	3	9
T2a	6	17
T2b	2	6
T3	7	21
T4	15	44
**N Stage (8th Edition)**		
N0	1	3
N1	4	12
N2	19	56
N3	10	29
**TNM Stage (8th Edition)**		
Stage IIB	2	6
Stage IIIA	11	32
Stage IIIB	13	38
Stage IIIC	8	24
**T Stage (9th Edition)**		
T1b	1	3
T1c	3	9
T2a	6	18
T2b	2	6
T3	7	20
T4	15	44
**N Stage (9th Edition)**		
N0	1	3
N1	4	12
N2a	9	26
N2b	10	29
N3	10	29
**TNM Stage (9th Edition)**		
Stage IIB	4	12
Stage IIIA	8	24
Stage IIIB	14	40
Stage IIIC	8	24

**Abbreviations:** PD-L1 = Programmed death-ligand 1.

**Table 2:** Univariate analysis of prognostic factors associated with overall survival and progression-free survival.

Covariates	2-y OS (%)	*p* Value	2-y PFS (%)	*p* Value
**Age (years)**		0.729		0.250
<65	72		81	
≥65	83		41	
**Gender**		0.418		0.965
Female	88		62	
Male	78		51	
**Smoking status**		0.840		0.696
Yes	83		57	
No	60		42	
**Histopathology**		0.532		0.787
Squamous cell carcinoma	87		54	
Adenocarcinoma	83		56	
Other	0		50	
**TNM Stage (9th Edition)**		0.123		0.167
Stage IIB	100		38	
Stage IIIA	86		43	
Stage IIIB	83		73	
Stage IIIC	64		27	
**Total Immunotherapy cycle**		0.250		0.207
<11	67		33	
≥11	93		69	
**The time interval between the diagnosis and RT initiation (months)**				
<4.46	77		60	
≥4.46	85	0.295	55	0.654
**Intent to RT**		0.881		0.320
Consolidation	82		63	
Progression	75			
**PD-L1 score (%)**		0.615		0.857
<20	90		55	
≥20	76		59	
**PIV**		0.220		0.121
<1381.5	85		61	
≥1381.5	69		34	

**Abbreviations:** RT = Radiotherapy; PD-L1 = Programmed death ligand 1; PIV = Pan-immune-inflammation-value. *p* < 0.05 was considered statistically significant.

## Data Availability

The original contributions presented in the study are included in the article, further inquiries can be directed to the corresponding author.
